# High-resolution structural-functional substrate-trigger characterization: Future roadmap for catheter ablation of ventricular tachycardia

**DOI:** 10.3389/fcvm.2023.1112980

**Published:** 2023-02-16

**Authors:** Job Stoks, Ben J. M. Hermans, Bas J. D. Boukens, Robert J. Holtackers, Suzanne Gommers, Yesim S. Kaya, Kevin Vernooy, Matthijs J. M. Cluitmans, Paul G. A. Volders, Rachel M. A. ter Bekke

**Affiliations:** ^1^Department of Cardiology, Cardiovascular Research Institute Maastricht (CARIM), Maastricht University Medical Center+, Maastricht, Netherlands; ^2^Department of Advanced Computing Sciences, Maastricht University, Maastricht, Netherlands; ^3^Biomedical Research Institute, Hasselt University, Diepenbeek, Belgium; ^4^Department of Physiology, Cardiovascular Research Institute Maastricht (CARIM), Maastricht University, Maastricht, Netherlands; ^5^Department of Medical Biology, Amsterdam University Medical Center (UMC), Amsterdam Medical Center (AMC), Amsterdam, Netherlands; ^6^Department of Radiology and Nuclear Medicine, Cardiovascular Research Institute Maastricht (CARIM), Maastricht University Medical Center+, Maastricht, Netherlands; ^7^Philips Research, Eindhoven, Netherlands

**Keywords:** VT, image integration, multi-modality, electroanatomical mapping, ECGI, CMR, personalized medicine, CT

## Abstract

**Introduction:**

Patients with ventricular tachyarrhythmias (VT) are at high risk of sudden cardiac death. When appropriate, catheter ablation is modestly effective, with relatively high VT recurrence and complication rates. Personalized models that incorporate imaging and computational approaches have advanced VT management. However, 3D patient-specific functional electrical information is typically not considered. We hypothesize that incorporating non-invasive 3D electrical and structural characterization in a patient-specific model improves VT-substrate recognition and ablation targeting.

**Materials and methods:**

In a 53-year-old male with ischemic cardiomyopathy and recurrent monomorphic VT, we built a structural-functional model based on high-resolution 3D late-gadolinium enhancement (LGE) cardiac magnetic resonance imaging (3D-LGE CMR), multi-detector computed tomography (CT), and electrocardiographic imaging (ECGI). Invasive data from high-density contact and pace mapping obtained during endocardial VT-substrate modification were also incorporated. The integrated 3D electro-anatomic model was analyzed off-line.

**Results:**

Merging the invasive voltage maps and 3D-LGE CMR endocardial geometry led to a mean Euclidean node-to-node distance of 5 ± 2 mm. Inferolateral and apical areas of low bipolar voltage (<1.5 mV) were associated with high 3D-LGE CMR signal intensity (>0.4) and with higher transmurality of fibrosis. Areas of functional conduction delay or block (evoked delayed potentials, EDPs) were in close proximity to 3D-LGE CMR-derived heterogeneous tissue corridors. ECGI pinpointed the epicardial VT exit at ∼10 mm from the endocardial site of origin, both juxtaposed to the distal ends of two heterogeneous tissue corridors in the inferobasal left ventricle. Radiofrequency ablation at the entrances of these corridors, eliminating all EDPs, and at the VT site of origin rendered the patient non-inducible and arrhythmia-free until the present day (20 months follow-up). Off-line analysis in our model uncovered dynamic electrical instability of the LV inferolateral heterogeneous scar region which set the stage for an evolving VT circuit.

**Discussion and conclusion:**

We developed a personalized 3D model that integrates high-resolution structural and electrical information and allows the investigation of their dynamic interaction during arrhythmia formation. This model enhances our mechanistic understanding of scar-related VT and provides an advanced, non-invasive roadmap for catheter ablation.

## 1. Introduction

Catheter ablation of arrhythmogenic substrate and triggers of ventricular tachycardia (VT) reduces arrhythmia burden and implantable cardioverter-defibrillator (ICD) shocks (1, 2) but recurrences [30–40% within 1–2 years (3, 4)], costs and complication rates remain high (5, 6). This limited procedural efficacy is attributed to the difficulty of identifying heterogeneous scar corridors related to the critical VT isthmus based on electrogram characteristics and pace mapping, especially during substrate-based ablation.

Recent advances in the field of catheter ablation for scar-related VT have primarily focused on defining target locations for ablation through pre-procedural characterization of the ventricular scar architecture by combining imaging techniques [such as computed tomography (CT) or cardiac magnetic resonance imaging (CMR)] with electrocardiogram (ECG)-VT algorithms. Image-guided or -aided substrate ablation may increase long-term success, decrease recurrence rates and reduce procedural duration (7, 8). Imaging-based approaches mostly focus on structural myocardial targets, ignoring 3D functional information such as activation/propagation maps, repolarization maps, wavefront direction or arrhythmia triggers. Electrocardiographic imaging (ECGI) combines body-surface potential mapping and anatomical imaging to provide a non-invasive reconstruction of electrical activity. It enables a beat-to-beat non-invasive 3D assessment of epicardial activation, repolarization and propagation, besides the pinpointing of the VT exit site with relatively high spatial and temporal resolution (9, 10).

In this study, we combined ECGI with high-resolution 3D dark-blood late gadolinium enhancement (3D-LGE) CMR and cardiac CT in a case with ischemic cardiomyopathy and recurrent monomorphic VT, providing a patient-specific structural-functional model to improve mechanistic understanding and to provide a road map for future personalized catheter ablation for VT. Additionally, we examined the accuracy of non-invasively determined parameters compared to standard invasive VT-substrate characteristics.

## 2. Materials and methods

We constructed a personalized 3D cardiac model that integrates the individualized structural-functional characteristics of this case to identify mechanisms of arrhythmia formation and to develop an individualized framework for future VT ablation guidance.

The model incorporates (1) 3D dark-blood LGE CMR and cardiac CT delineating the myocardial anatomy and scar (structural aspects); and (2) functional-electrical aspects by non-invasive ECGI. Model outcomes were compared to data from invasive electro-anatomical mapping (EAM), and arrhythmia mechanisms were investigated.

### 2.1. Patient characteristics

**FIGURE 1:**
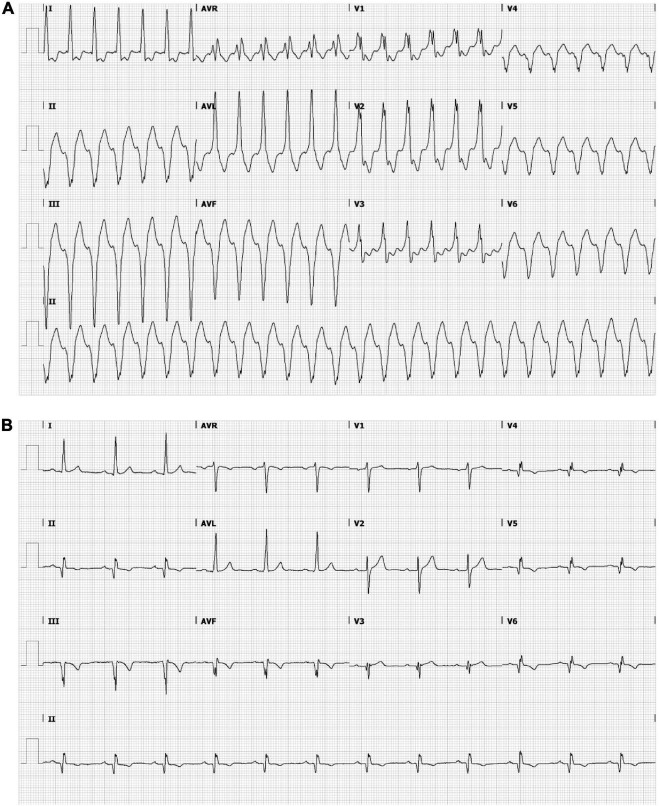
Ensure that all the figure captions are correct, and that all figures are of the highest quality/resolution. If necessary, you may upload improved figures to the Production Forum. Please note that figures must be cited sequentially, per the author guidelines. The patient’s electrocardiogram during ventricular tachycardia (VT) **(A)** and sinus rhythm **(B)**. **(A)** Monomorphic VT demonstrating a wide QRS complex of 151 bpm with a right bundle branch block pattern (transition in V3) and a superior axis. The positive polarities in leads I and aVL suggest an inferomedial to inferobasal, septal origin (47). **(B)** Sinus rhythm, 72 bpm, left axis deviation, a QRS width of 112 ms, pathological Q waves in inferior leads and V3–V6 and negative T waves in inferior leads and in V4–V6. Q waves are indicative of previous inferior/apical infarctions.

A 53-year-old male was admitted to our hospital for electrical storm due to recurrent hemodynamically tolerated, sustained monomorphic VTs with an inferobasal to mid, septal left-ventricular (LV) origin (Figure 1A) (11). He was previously known with anterior and inferolateral myocardial infarctions (LV ejection fraction 31%), also evident from the 12-lead electrocardiogram (Figure 1B), and coronary artery bypass grafting. The VT was managed with intravenous infusion of amiodarone. A 3D-LGE CMR and cardiac CT scan were performed to aid catheter ablation for VT. Additional electrical information was obtained by body-surface potential mapping before and during the VT ablation procedure.

### 2.2. A 3D dark-blood late gadolinium-enhancement CMR

The patient underwent a free-breathing 3D-LGE CMR with high isotropic resolution (acquired resolution 1.6 mm × 1.6 mm × 1.6 mm, reconstructed 0.8 mm × 0.8 mm × 0.8 mm) and dark-blood nulling (12) on a 1.5 T MR system (Ingenia, Philips Healthcare, Best, Netherlands). This allowed for more accurate scar demarcation and image quality with respect to conventional bright-blood nulled LGE CMR (13). Details of the dark-blood LGE mechanism without using additional magnetization preparation has were described earlier (14).

Cardiac magnetic resonance-image processing was performed using a research software tool (ADAS3D Medical SL, Barcelona, Spain) (15). Pixel-signal intensity (PSI) maps were created from the 3D delayed enhancement acquisitions, and normalized for maximal PSI. A normalized PSI-based cut-off above 0.6 was defined as dense scar; border zone tissues were allocated in case of values between 0.4 and 0.6% (16). The LV myocardium was divided in nine three-dimensional transmural layers color-coded for scar architecture. Heterogeneous tissue corridors, defined as continuous corridors of border zone tissue connecting two regions of healthy tissue, bordered by dense scar and/or an anatomical barrier, were automatically calculated and visualized on the shells by the research tool (15). Subsequently, 3D-LGE CMR scar transmurality was defined as the percentage of transmural layers having either dense scar (weight 1) or border zone (weight 0.5). Scar transmurality was projected onto the endocardial shell.

### 2.3. Computed tomography and electrocardiographic imaging

Prior to the VT ablation procedure, an ECG-gated helical dual-source CT scan (Somatom Force, Siemens Healthineers, Forchheim, Germany) using intravenous iodine contrast medium was performed during breath-hold, to visualize cardiac geometry. Additionally, the patient underwent a low-dose thoracic CT scan to locate the body-surface electrodes, required for ECGI.

The ECGI was performed as previously described and validated (17, 18). Briefly, 224 Ag-AgCl electrodes (BioSemi, Amsterdam, The Netherlands) were attached to the patient’s torso to record body-surface potentials at a 2048 Hz sampling rate starting 20 min prior to, and during, the electrophysiological study. From the thoracic CT scan, a 224-electrode torso was manually digitized. A ∼2000-node diastolic cardiac geometry was semi-automatically segmented (19). Body-surface potential measurements were performed during sinus rhythm, atrial pacing, during programed ventricular stimulation for VT induction and for evoked delayed potential (EDP) identification (20), and during VT.

Epicardial ventricular unipolar electrograms (UEGs) were reconstructed for selected beats through previously detailed methods (17). For each UEG, activation times (ATs) were determined from the steepest downslope (of the UEG QRS complex); this was done with a spatiotemporal approach that considers the spatial flow of current (21). ATs of each beat were calculated with respect to the average of the first 25 epicardial activations according to ECGI. UEGs were disregarded at the valvular base of the ventricles. VT dynamics were assessed by calculating the absolute activation time differences between consecutive VT beats.

### 2.4. Catheter ablation procedure for VT

The patient underwent continued body-surface potential mapping during the VT ablation procedure (Supplementary Figure 1). The procedure was performed under general anesthesia without preemptive hemodynamic support. The CARTO navigation system (Biosense Webster Inc., Diamond Bar, CA, USA) was used for guidance of the procedure. Both retrograde and anterograde access were obtained. The ADAS3D-derived anatomy and scar architecture were merged with the aortic root, main stem and pulmonary veins. After positioning of a quadripolar diagnostic catheter (6 F, CRD-2™, Abbott, Chicago, IL, USA) in the right-ventricular (RV) apex, programed RV stimulation was performed at three driving cycle lengths (600, 500, and 400 ms) with up to three ventricular extrastimuli (≥200 ms) to induce sustained VT (>30 s or necessitating termination because of hemodynamic instability) and to determine the RV effective refractory period (ERP). During right-atrial (RA) pacing, a detailed electroanatomical voltage map was obtained using a high-density mapping (PentaRay, Biosense Webster Inc., Diamond Bar, CA, USA) and an irrigated contact-force sensing 3.5 mm-tip catheter (SmartTouch, Biosense Webster Inc., Diamond Bar, CA, USA). Near-field peak-to-peak bipolar voltage amplitude below 1.5 mV indicated scar, with dense scar <0.5 mV and border zone 0.5–1.5 mV. EDPs, characterized by low-amplitude near-field potentials with 10-ms delay or block after RV extrastimulus (S2 50 ms above the ERP), were tagged in the 3D system, as previously described (22). The VT site-of-origin was identified using pace mapping aimed at a mean correlation coefficient between the VT-QRS morphology and the paced-QRS of >90% (23). Substrate modification was performed aiming at complete EDP elimination and targeting the VT site-of-origin using radiofrequency energy delivery (power 50 W, temperature limit 43°C, flow rate 20–30 mL/min). After the last lesion set, the substrate was reassessed for the abolition of EDPs, and programed ventricular stimulation was repeated to test for VT inducibility.

### 2.5. Postprocedural image integration and analysis

The geometries from 3D-LGE CMR, CT, ECGI and invasive EAM were manually aligned and registered digitally in 3D Slicer (24). The most important anatomic landmarks used for integration were: the LV and RV epicardium, LV apex and aorta from CT; LV apex, epicardium, endocardium and aorta from 3D-LGE CMR; and LV endocardium, apex and aorta from EAM. The accuracy of the image integration was based on Euclidean node-to-node distance calculation and visual evaluation. The EAM geometry was converted isotropic (i.e., equal node-to-node distances throughout the geometry) to ensure that all regions of the heart had equal weight in downstream analyses. Further analyses were performed in MATLAB R2020b (MathWorks, Natick, MA, USA).

Information from 3D-LGE CMR and ECGI was combined to examine triggers, substrate, and their interactions. 3D-LGE CMR scar delineation was compared to areas of low invasive bipolar voltage, by finding the corresponding nearest neighbors of the CMR anatomy and the invasive electroanatomic anatomy. Any neighbors with a corresponding distance of >10 mm from each other were disregarded. The colocalization of endocardial EDPs with MRI-derived heterogeneous tissue corridors was also evaluated, disregarding EDPs that were tagged >10 mm from the endocardial 3D-LGE CMR shell.

## 3. Results

### 3.1. Ablation procedure and outcome

A sustained monomorphic VT arising from the inferobasal LV, closely resembling the documented arrhythmia, was repeatedly induced by programed electrical stimulation (by minimally two ventricular premature beats) and led to hemodynamic compromise. The high-density LV-endocardial bipolar voltage map (5,375 points) revealed two remote areas of scar in the lateral and apical regions of the LV, colocalizing with areas of increased endocardial PSI based on 3D-LGE CMR. 21 EDPs were tagged. Pace mapping at the inferobasal LV resulted in a 92% match with the induced VT. Radiofrequency delivery targeted at the inferolateral EDPs and the site of VT origin, eliminated the EDPs and rendered the patient non-inducible at the end of the procedure. Total procedural time was 330 min; fluoroscopy time 10 min. The procedure was without any complications. A single-chamber ICD was implanted afterward. No VT had recurred at 20 months follow-up.

### 3.2. Contrast-enhanced CT and 3D dark-blood late gadolinium-enhancement CMR

**FIGURE 2:**
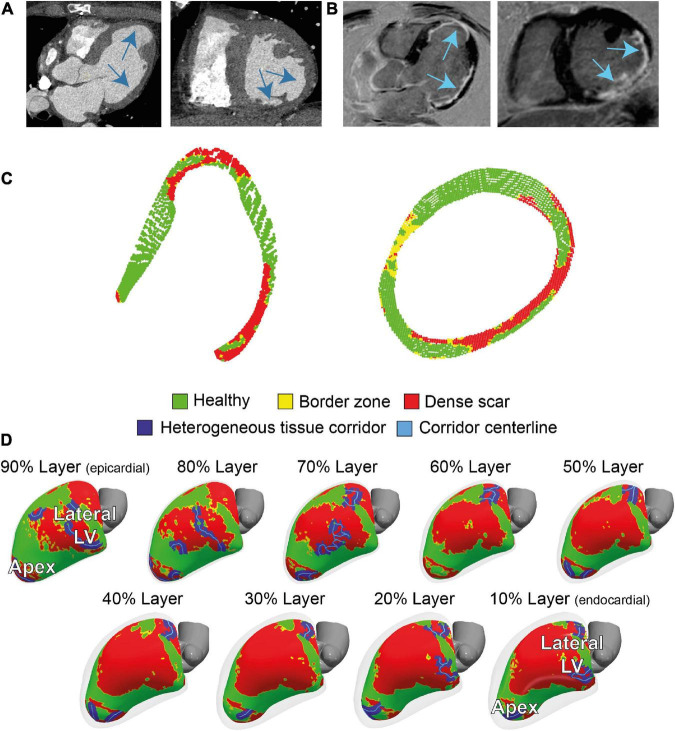
Cardiac imaging of the patient prior to the ablation procedure. **(A)** Contrast-enhanced dual-source computed tomography (CT), performed for anatomical reference showing wall thinning in the apical aneurysm and laterobasal aspect of the left ventricle (LV). **(B)** 3D dark-blood late gadolinium enhancement cardiac magnetic resonance imaging (LGE CMR) LGE CMR showing areas with hyperenhancement in the laterobasal and apical aspects of the LV. **(C)** Two cross-sectional views of LV scar, detected by 3D dark-blood LGE CMR. **(D)** CMR-derived healthy and scar tissue, including corridors and their centerlines, depicted in nine transmural layers (interlayer distance approximately 0.8 mm) of the ventricular myocardium. Colors correspond to colors in panel **(C)**.

Contrast-enhanced multidetector CT showed wall thinning in the inferolateral LV and apex (Figure 2A and Supplementary Figure 2). The CMR revealed a partly transmural infarcted area in the inferolateral and apical segments with aneurysm formation (Figure 2B and Supplementary Videos 1, 2). Dense scar and border zone, as detected through CMR, consisted of non-uniform transmural patterns (Figure 2C). Heterogeneous tissue corridors, identified from CMR (Figure 2D) were localized in the basal to mid (infero) lateral, and apical aspects of the LV.

**FIGURE 3:**
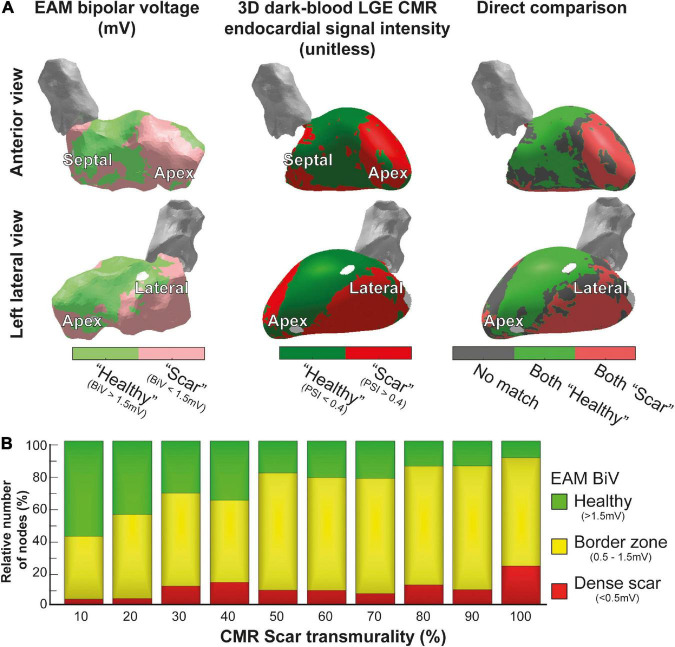
Comparison of 3D-late gadolinium enhancement cardiac magnetic resonance imaging (LGE CMR) to electroanatomical mapping (EAM). **(A)** Invasive EAM bipolar voltage map (left) and 3D dark-blood LGE CMR endocardial signal intensity (middle), and the comparison of both (right). Areas of agreement regarding healthy tissue are shown in green, agreement regarding scar tissue are shown in red, and differences between both maps are shown in gray. **(B)** CMR scar transmurality compared to EAM bipolar voltage. Higher transmurality of scar (detected by 3D-LGE CMR) was associated with lower bipolar voltage in EAM.

Manual image integration of invasive EAM and 3D-LGE CMR endocardial geometry led to a mean Euclidean node-to-node distance 5 ± 2 mm. Increased PSI (>0.4) of the innermost endocardial layer of 3D dark-blood LGE CMR closely resembled reduced endocardial bipolar voltage (<1.5 mV) using previously defined cutoffs (15), while areas of non-agreement were near the scar-to-healthy border (Figure 3A). Both modalities agreed (both “scar” or both “healthy”) for 68% of tissue, and disagreed for the remaining 32%, mostly concerning the border zone. According to both modalities, the majority of scar consisted of border zone (76% according to EAM, 85% according to 3D-LGE CMR). Predicting bipolar scar through the CMR endocardial signal intensity rendered a receiver operating characteristic (ROC) area under the curve of 0.72. Further comparison of 3D-LGE CMR scar transmurality to EAM bipolar voltage showed that low voltages were associated with higher scar transmurality, and vice versa (Figure 3B).

**FIGURE 4:**
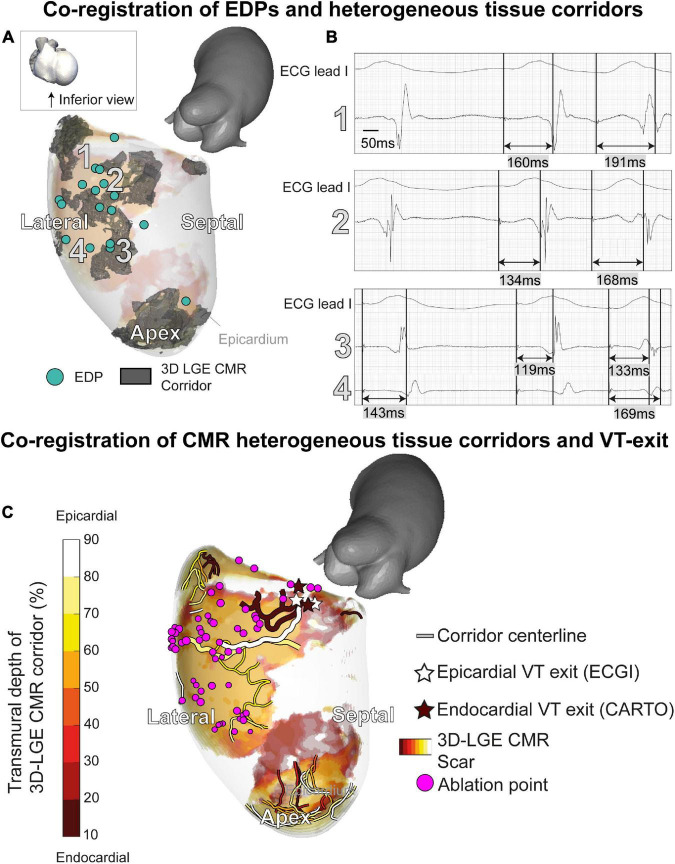
A 3D-late gadolinium enhancement cardiac magnetic resonance imaging (LGE CMR) heterogeneous tissue corridors and evoked delayed potentials (EDPs). **(A)** Annotation of EDPs during the electroanatomical mapping (EAM) procedure, compared to a superimposed anatomical view of transmural heterogeneous tissue corridors detected through 3D LGE CMR. All EDPs except one coincided with ventricular scar or border zone. EDPs qualitatively coincided well with corridors close to the endocardium. **(B)** EDPs on locations 1–4 in panel A. **(C)** Investigating of ventricular tachycardia (VT) working mechanisms through structural-functional image overlay. Transmural layers of scar and border zone with centerlines of slow-conducting heterogeneous tissue corridors are superimposed and colored. VT exits were determined by pace mapping during the procedure. Invasively determined endocardial (brown) and non-invasively determined epicardial (white) exits coincide at the end of epicardial and endocardial corridors. Corridors of interest leading to the VT exit have an increased width. Ablation of the substrate (purple), including the identified corridors, rendered the patient arrhythmia-free.

A total of 17/21 EDPs were within 10 mm of the endocardial CMR-wall. 15/17 (88%) of these EDPs were located in an area of endocardial scar detected by 3D dark-blood LGE CMR (Figures 4A, B). EDPs were often identified within or close to (transmural) CMR-derived corridors. When projecting all EDPs and corridors onto the endocardial CMR layer, the average Euclidean distance from EDP to the nearest corridor was 3 (0–6) mm [median (first and third quartiles)].

### 3.3. Structural-functional substrate characterization

Structural-functional overlay of the 3D-LGE CMR and ECGI data (body-surface potential mapping during the ablation procedure) allowed the investigation of the VT trajectory and mechanism. In Figure 4C, the endocardial (invasive EAM) and epicardial (ECGI) locations of the VT exit according to pace mapping are shown, together with transmural fibrosis and PSI-derived heterogeneous tissue corridors. ECGI pinpointed the VT exit in close proximity (spatial accuracy 10/14/13 mm) to the invasive endocardial site with the highest pace-mapping agreement (0.90/0.92/0.92, respectively). When projecting the ECGI-exit onto the endocardial geometry, i.e., omitting the endocardial-to-epicardial distance, the distance between invasive and non-invasive VT exit decreased to 5/12/12 mm, respectively. Both epicardial and endocardial heterogeneous tissue corridors were adjacent to the VT exit, possibly serving as slow-conducting channels (isthmuses) for the VT. Radiofrequency ablation to, amongst other sites, the entrance of these corridors, rendered the patient arrhythmia-free (20 months follow-up).

**FIGURE 5:**
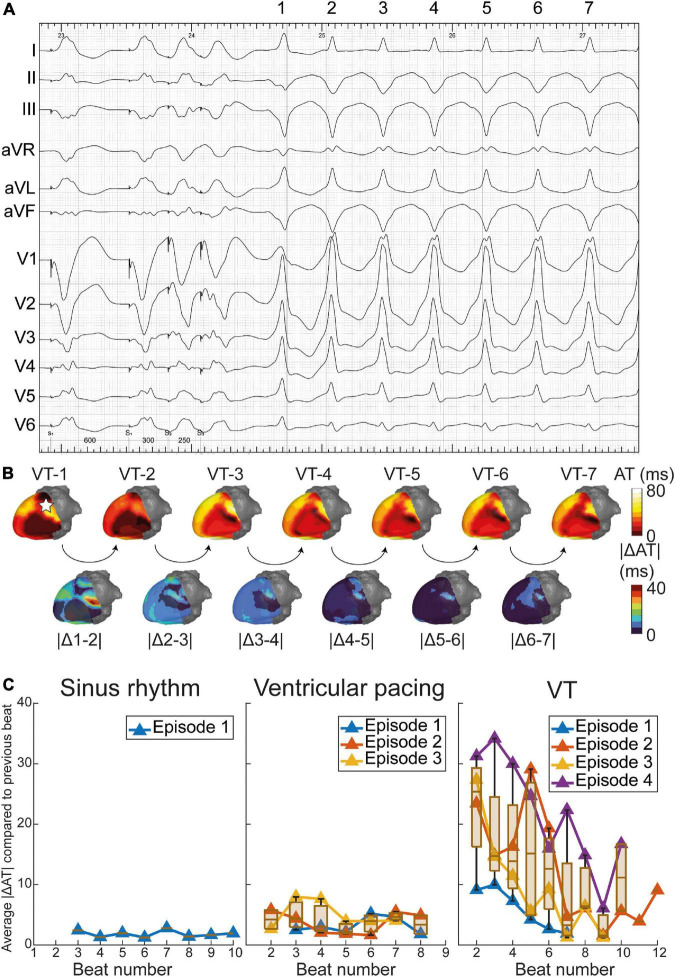
Dynamics of epicardial activation patterns through electrocardiographic imaging (ECGI). **(A)** ventricular tachycardia (VT) during procedure in which beats 1 through 7 are depicted. **(B)** Activation patterns of VT beats 1 through 7, with absolute difference in activation time (AT) of consecutive beats shown below. The pentagram in VT-1 illustrates the origin of the premature ventricular complex (PVC) (Supplementary Figure 3). For reference, dynamics of activation patterns in sinus rhythm (SR) and pacing are also shown. **(C)** Average absolute difference in activation times of consecutive beats, over the entire epicardium. Dynamics in activation patterns decrease over time for several episodes (inductions) of VT, while SR and ventricular pacing show stable activation patterns.

Although the monomorphic VT (with exception of its first beat) appeared stable on the ECG (Figure 5A), ECGI revealed significant beat-to-beat differences in activation patterns during the onset of VT (Figure 5B). The VT stabilized over time, causing beat-to-beat differences in its activation pattern to decline. The anterolateral area of early activation migrated inferolaterally as the VT evolved. This area of shifting early activation correlated with CMR-detected corridors leading to the VT exit (Figure 4B). Beat-to-beat-differences in activation pattern were present during all inductions (episodes) of VT, higher than during sinus rhythm and ventricular pacing (Figure 5C).

Prior to the electrophysiological study, a premature ventricular complex (PVC) was recorded with ECGI (Supplementary Figure 3A). The PVC origin matched significantly, although not completely, with the VT exit. ECGI pinpointed the PVC to the lateral LV wall (Supplementary Figure 3B). The crowded activation isochrones superior to the first-activated area suggest a conduction block in the basolateral LV (blue arrow). CMR-detected heterogeneous tissue corridors (numbers 1–3, Supplementary Figure 3C) connected the PVC and VT origins and coincided with the area of shifting early activation during VT (Supplementary Figure 3C). Hence, we hypothesize the PVC could be involved in VT initiation.

## 4. Discussion

In this proof-of-concept study, we developed a 3D structural-functional model for scar-related VT by combining 3D dark-blood LGE CMR and ECGI (of PVC and VT) with high spatial and temporal resolution. This model was developed in an exemplary case with recurrent monomorphic VT in the setting of ischemic cardiomyopathy. Patient-specific VT substrates, triggers, and their interactions were detailed, in a non-invasive manner, serving as a roadmap for future individualized ablation strategies. Integrating high-density invasive EAM advanced the understanding of possible VT mechanisms.

In our integrated model, the (invasive and non-invasive) VT exit colocalized at the end of CMR-detected heterogeneous tissue corridors. Furthermore, the patient’s spontaneous PVC originated in close proximity to the VT exit and CMR-detected corridors. This suggests that the PVC may be involved in patient’s VT formation. Moreover, EDPs were found close to heterogeneous tissue corridors, suggesting a strong link between functional endocardial conduction slowing/block and structural border zone channels.

### 4.1. 3D dark-blood LGE CMR

Acquiring 3D-LGE CMR with high isotropic resolution visualizes myocardial fibrosis from every desired direction compared to a fixed viewing angle when using (non-isotropic) 3D or standard 2D imaging (25). Subtle scar architectures, such as submillimeter tissue corridors, may be missed using these latter acquisition techniques. Additionally, the dark-blood nulling CMR, recently validated against histopathology in an experimental large-animal model (13), allows for improved detection and quantification of (sub) endocardial fibrosis, often encountered in ischemic etiologies, compared to conventional bright-blood LGE (14). We have shown that 3D dark-blood LGE CMR has a reasonable correlation, at least qualitatively, with the endocardial bipolar voltage. We found a 68% agreement between reduced (<1.5 mV) endocardial bipolar voltage and increased (>0.4) 3D-LGE CMR-signal intensity when applying a point-by-point comparison between both modalities. Areas of non-agreement were predominantly found at the healthy-to-scar-border zone. The ROC area under the curve to define areas of low voltage through 3D-LGE CMR was 0.72, which compared with the low range of values found in a recent study, although different methods of analysis may have affected the results (26). The observed electrostructural mismatch stems from the fundamentally different assessment of scar characteristics, with varying spatial resolution and dissimilar gating for cardiac phases and respiratory motion, besides the variable impact of modulators (e.g., contact force, fiber orientation, wavefront direction, and sympathetic hyperinnervation). The scar border zone may be especially susceptible to these factors.

Additionally, we have shown a good correlation between the 3D dark-blood LGE CMR-derived myocardial heterogeneous tissue corridors and the invasively measured areas of functional electrical conduction slowing or block (15). These corridors may comprise slow-conducting pathways, or protected isthmuses, that may facilitate reentrant excitation (15) upon unidirectional conduction delay or block (22).

### 4.2. ECGI

ECGI has been validated in multiple studies. For a recent overview we refer to Stoks and Cluitmans (27). Its strength lies in the reconstruction of (paced) rhythm of single origin (28). In a structurally normal heart, ECGI was more accurate than the 12-lead ECG to pinpoint the origin of VT or PVC (29). The 12-lead ECG was even less precise in the presence of myocardial scarring (30). We have shown that ECGI-based reconstructions localized the site of ventricular activation (adjacent to scar border zone) by pace mapping at ∼10 mm from the endocardial location (if endocardial-to-epicardial distance was omitted). This was more accurate than in a recent study using a commercial system (30) but comparable to other reports (27).

Moreover, ECGI can generate a complete 3D-epicardial activation map in a single beat, while several VT cycle lengths are needed during invasive contact mapping EAM to construct an activation or propagation map (which is often not possible since VT is often polymorphic or hemodynamically not tolerated). This may be relevant for investigating the dynamic behavior of VT, especially in polymorphic VT but also during the onset of monomorphic VTs. In our case, ECGI uncovered marked initial activation dynamicity, only stabilizing after several beats. An area of electrical instability (shifting early activation) during VT was present close to the VT exit and structural-functional corridors (Figures 4C, 5B).

### 4.2. Structural-functional VT substrate characterization

**FIGURE 6:**
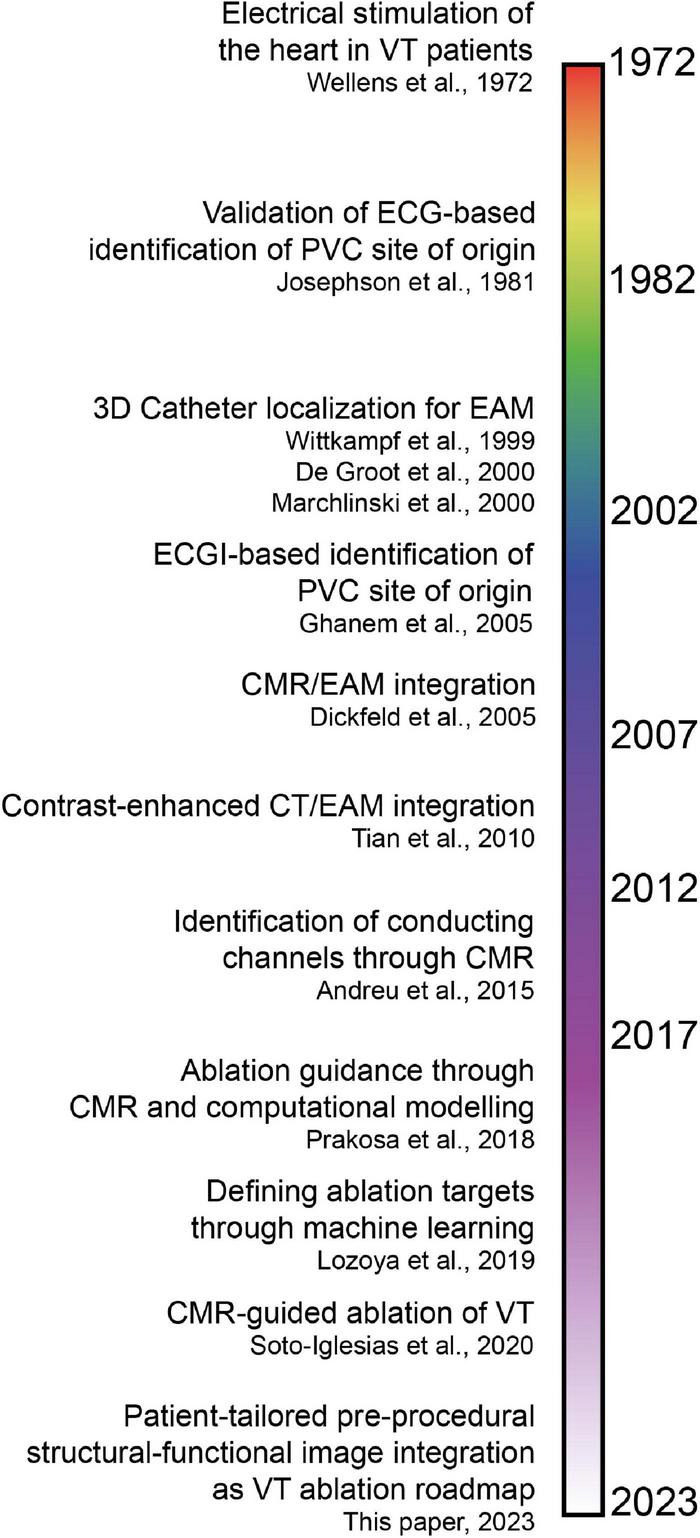
Timeline of invasive and non-invasive techniques to identify critical components of the ventricular tachycardia (VT) circuit and guide catheter ablation (7, 15, 31–33, 35–38, 43, 44). Note that the time axis is not linear, which is reflected by the color gradients. ECG, electrocardiogram; PVC, premature ventricular complex; EAM, electroanatomical mapping; ECGI, electrocardiographic imaging; CMR, cardiac magnetic resonance imaging; CT, computed tomography.

Non-invasive modalities identifying critical components of the VT circuit to guide catheter ablation have evolved considerably over the last decades. Historical milestones in the human heart are shown in Figure 6.

In 1972, Wellens et al. found that, in (mostly) post-infarction patients, VTs were based on reentrant excitation, that could be terminated through electrical stimulation (31). In 1981, Josephson et al. compared 12-lead ECG QRS morphologies of VTs to endocardial VT exit sites in 34 patients (32), demonstrating that the presence of myocardial scar diminishes the ECG accuracy to pinpoint the endocardial VT exit.

In 1999, Wittkampf et al. first validated an impedance-based 3D catheter navigation system in humans for EAM (33). In 2000, De Groot et al. introduced a 3D-catheter localization system using ultrasound transducers to guide VT ablation (34), that allowed real-time display of the catheter tip and repositioning to previously marked sites. In the same year, Marchlinski et al. introduced the magnetic mapping-based CARTO system to perform linear substrate ablation which proved effective to significantly reduce VT recurrence (35). These 3D-navigation systems could be considered the foundation of the modern-day EAM.

In 2005, pioneering work on image integration was done by Dickfeld et al. who coregistered MRI acquisitions with the electromagnetic catheter positioning system to guide catheter navigation in the human right atrium and ventricle (36). In the same year, Ghanem et al. showed that near-simultaneous ECGI-derived (non-invasive) electrograms had a moderate correlation with invasive potentials recorded in sinus rhythm and during endocardial and epicardial pacing in patients (37). Five years later, Tian et al. (38) coregistered contrast-enhanced CT relying on distinct anatomic, dynamic and perfusion characteristics indicative of myocardial scar and border zone with electrogram voltage obtained during endocardial point-by-point mapping. A high segmental accuracy was reported. Though contrast-enhanced CMR still has superior contrast resolution compared to CT, the latter is more easily applicable for scar demarcation in patients with an ICD and allows for increased temporal and spatial resolution (38). Furthermore, wall thickness analysis with CT accurately predicted isthmuses in postinfarction VT (39).

In 2008, Codreanu et al. performed image integration of EAM and LGE CMR to conclude that transmural scar (by CMR) could be detected by spiky electrograms, reduced voltage amplitudes or prolonged bipolar electrogram durations (40). A year later, Desjardins et al. investigated the relationship between reduced bipolar voltage in EAM and automatically segmented scar in LGE CMR, to find that sites critical to VT in humans are located within areas of delayed enhancement in CMR. In 2011, Wijnmaalen et al. performed a head-to-head comparison of LGE CMR with electroanatomical voltage mapping through image integration. They found that increased scar transmurality led to decreased endocardial bipolar voltage and that non-transmural and border zone scar was undetected by EAM (41). The combination of LGE CMR with multidetector CT is superior for structure-function relationship determination in scar-related VT (42). With the availability of 3D-LGE CMR acquisitions with high isotropic resolution, Andreu et al. (15) identified heterogeneous tissue corridors that colocalize in 79% with invasively recorded conduction channels (2015). VT ablation procedures guided by CMR led to increased procedure success, and decreased VT recurrence, inducibility and procedure time (7). In recent years, promising computational approaches have arisen. For example, Prakosa et al. performed CMR-based computational electrophysiological modeling, which allowed for prospective ablation guidance in a small cohort (43). Moreover, Lozova et al. used machine-learning algorithms to pinpoint ablation targets (44).

The aforementioned advances in the field of catheter-based ablation of scar-related VT have primarily focused on defining target locations for ablation through analysis of structural imaging modalities, typically CMR (43, 44) and/or CT (42). Though such structural detailing holds promise and allows to non-invasively investigate the substrate and perform virtual stress tests, these strategies usually do not consider patient-specific 3D functional electrical parameters. Our current proof-of-concept patient-tailored model encompasses 3D-functional and structural information and their interactions. As an example, we have shown that invasively- and non-invasively determined VT exits align colocalize with endings of heterogeneous tissue corridors, and are connected with the PVC site of origin. Moreover, we have demonstrated dynamic electrical instability of the heterogeneous scar region where the scar heterogeneity set the stage for an evolving VT circuit.

Combined, we argue that the integration (during spontaneous or induced PVCs and VT, including their spatiotemporal dynamics) of ECGI with high resolution 3D-LGE dark-blood CMR may be synergistic for the development of (non-invasive) individualized VT models to improve our understanding of VT mechanisms and treatment.

### 4.3. Future perspectives

Prospective studies are needed to investigate the procedural efficacy and cost-effectiveness of such personalized structural-functional models. This would require optimization of the modeling pipeline, including automated segmentation and merging of different modalities and automation of analyses, which is currently being developed.

Ideally, in future cases, ECGI recordings should be performed pre-procedurally during spontaneous VT or non-invasive programed stimulation (in the absence of deep sedation or general anesthesia) to allow preprocedural structural-functional analysis of the VT trajectory and its mechanism. This would guide targeted RF delivery to critical components of the VT circuit without the need for extensive intraprocedural voltage and pace mapping. For instance, CMR-derived heterogeneous tissue corridors (aligning to EDPs) connecting to ECGI-derived VT exit sites could be ablated. ECGI activation maps should be compared to invasively acquired (endo- and epicardial) VT activation/propagation maps to investigate excitation accuracy, VT dynamicity and trajectories.

In absence of an ICD, CMR may replace CT for localization of ECGI electrodes and the heart geometry, thereby omitting radiation. Beyond ischemic cardiomyopathies, this model could be extended other cardiac diseases with increased susceptibility for VT/VF, including non-ischemic and idiopathic etiologies. In the latter, ECGI uncovered previously undetected functional repolarization abnormalities, crucial for arrhythmia formation (18). Next, the model could be improved by the incorporation of static and dynamic ventricular repolarization. Furthermore, integration of our model with existing computational frameworks (digital twinning) may aid in identifying ablation targets and predict VT recurrences (risk stratification). Finally, structural-functional image modeling may be of value in novel emerging non-invasive ablation strategies, such as stereotactic cardiac radioablation (45, 46).

### 4.4. Study limitations

This was a proof-of-concept study based on one exemplary case. We aimed to first develop the model before recruiting more patients, which is currently ongoing. Firstly, although the ECGI VT activation map and exit site were acquired during the procedure, the study data were merged and analyzed retrospectively. The VT ablation procedure was not guided by our model. Secondly, anatomies were merged based on visual inspection. Advanced merging methods may further improve results of invasive EAM vs. ECGI/3D-LGE CMR comparisons (26). Thirdly, we used an epicardial-only formulation of ECGI (17) which does not actively consider *transmural* fibrosis, although this method is most widely applied and the most extensively validated (27). Lastly, we used bright-blood validated LGE CMR thresholds for scar delineation in the ADAS3D program. However, upon further analysis, in this patient, altering this threshold affected EAM vs. CMR scar comparisons by <1%.

## 5. Conclusion

In this proof-of-concept study, we combined 3D-LGE CMR, CT and ECGI imaging modalities to develop a patient-tailored high-resolution non-invasive structural-functional model to investigate VT substrate and trigger in a case of recurrent VT in the setting of ischemic cardiomyopathy. The model accurately pinpointed the VT exit site, collocated areas of structural and functional corridors and provided insight into scar transmurality and endocardial voltage characteristics. Beyond existing methods, our model encompasses beat-by-beat functional electrical information, such as dynamic 3D activation patterns of spontaneous ventricular ectopics and the VT circuit.

## Data availability statement

The raw data supporting the conclusions of this article will be made available by the authors, without undue reservation.

## Ethics statement

The studies involving human participants were reviewed and approved by the METC azM/UM. The patients/participants provided their written informed consent to participate in this study.

## Author contributions

JS, MC, PV, and RTB: study setup. JS: ECGI data acquisition and processing, data integration and analysis, and manuscript draft. YK: CT wall thickness analysis. RH and SG: CMR data acquisition. RTB: EAM and clinical data acquisition. JS, MC, BH, BB, PV, and RTB: conceptualization. MC, BH, RH, SG, YK, KV, BB, PV, and RTB: critical review of manuscript. All authors have read and approved the final manuscript and data interpretation.
