# Supplementary material for: High-resolution structural-functional substrate-trigger characterization: Future roadmap for catheter ablation of ventricular tachycardia
Source: Front Cardiovasc Med. 2023 Feb 16;10:1112980. doi: 10.3389/fcvm.2023.1112980 (PMC9978225; doi:10.3389/fcvm.2023.1112980)
Supplement: Supplementary file 1 [file Data_Sheet_1.DOCX]

Supplementary Material

# Supplementary Data


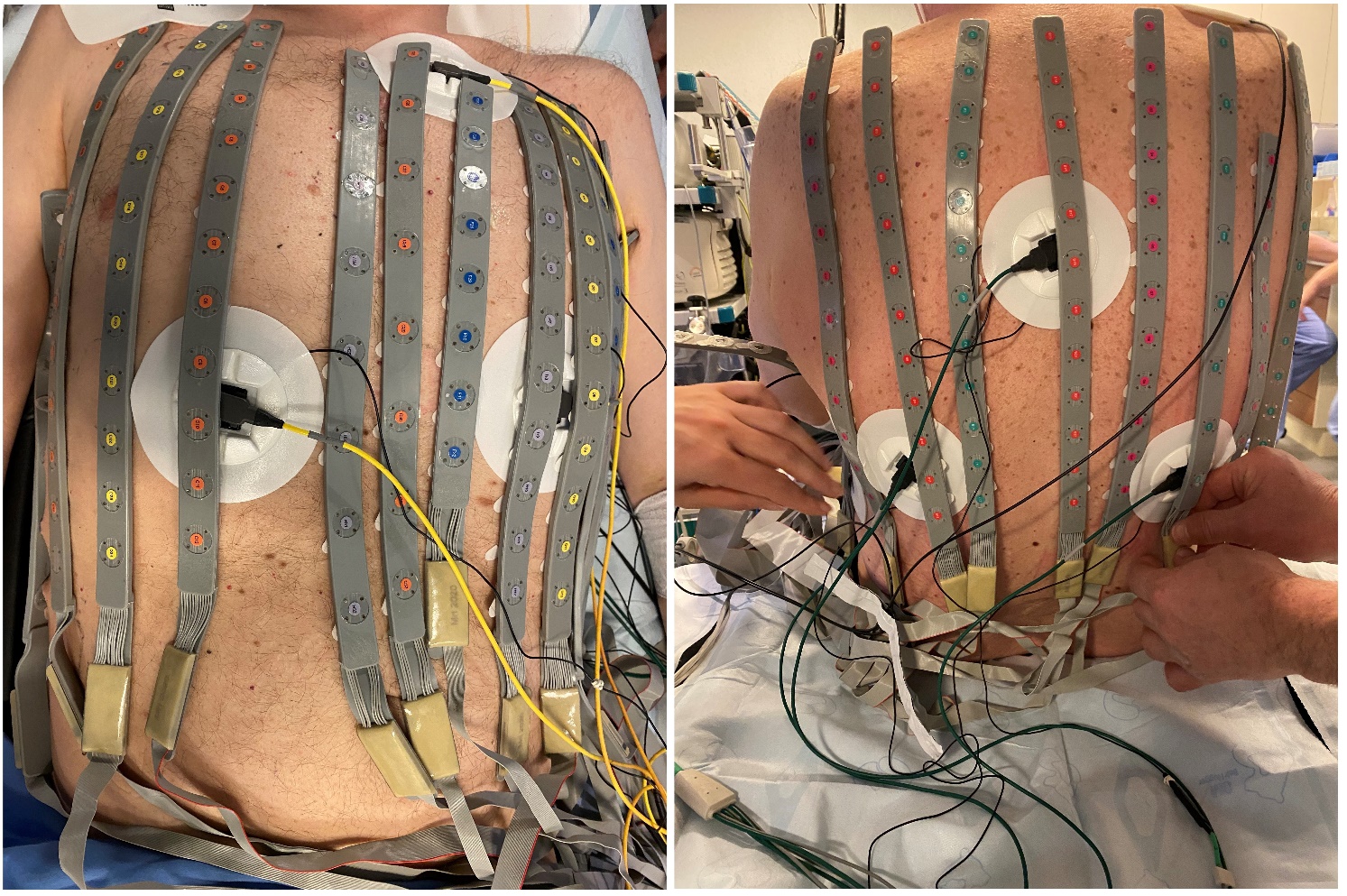
**Supplementary Figure 1.** Simultaneous body-surface potential mapping and electroanatomical mapping during ventricular tachycardia (VT) ablation procedure.

**
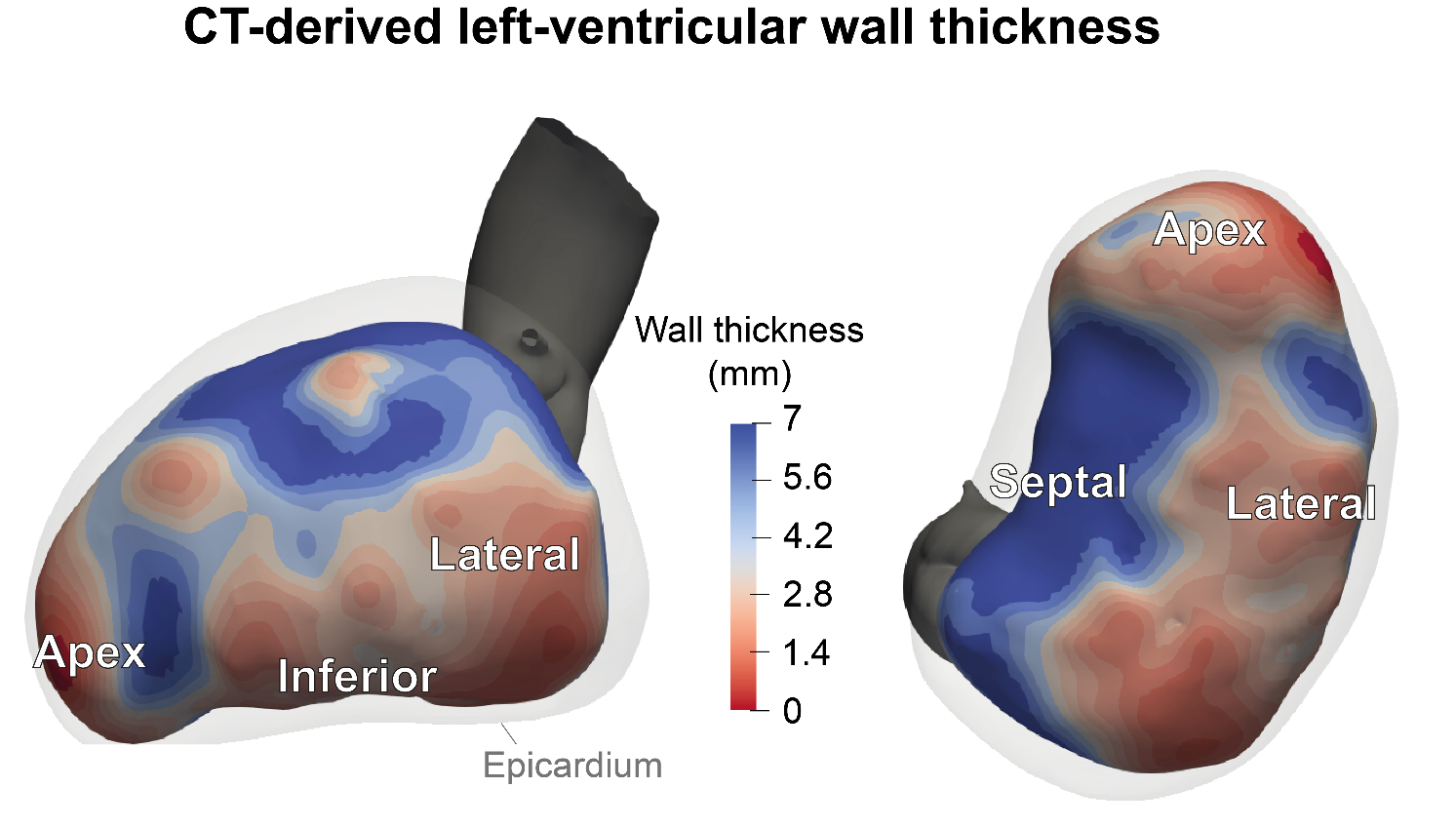
Supplementary Figure 2.** Left-ventricular (LV) wall thickness analysis through contrast-enhanced multidetector computed tomography (CT). The basal inferolateral and apical aspects of the LV show wall thinning.


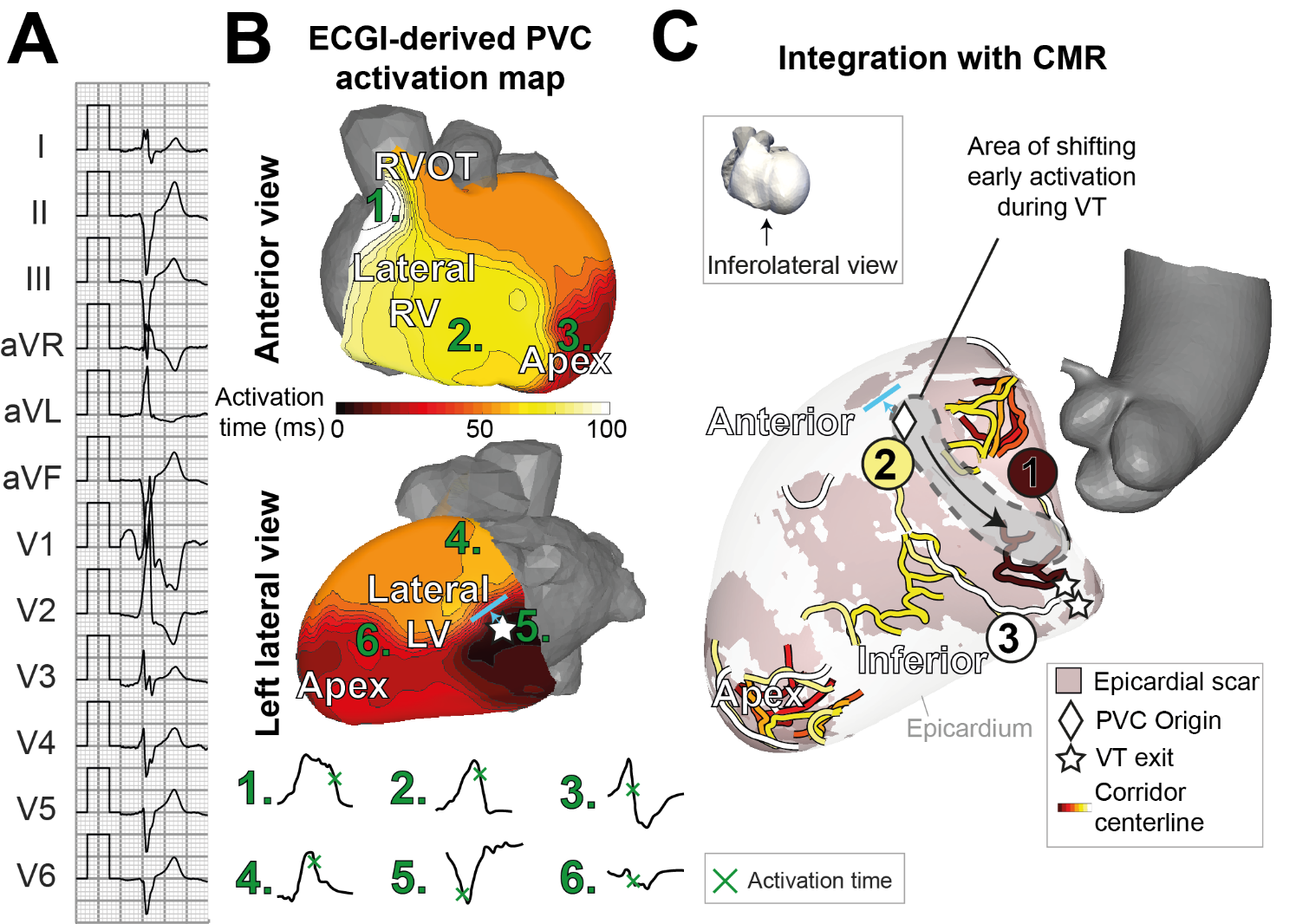


**Supplementary Figure 3.** Hypothesis on trigger-substrate interaction. Electrocardiographic imaging (ECGI) and integration with 3D dark-blood late gadolinium enhancement cardiac magnetic resonance imaging (LGE CMR). A) 12-Lead electrocardiogram (ECG) of premature ventricular complex (PVC). B) Activation map of this PVC through ECGI. The crowded activation isochrones in the left lateral view, depicted in gray, suggest conduction block. C) Structural-functional image overlay of 3D dark-blood LGE CMR (Figure 4) and ECGI-derived activation map during PVC (B). 1, 2 and 3 indicate possible heterogeneous tissue corridors through which the ventricular tachycardia (VT) could have travelled to reach the exit site.
